# Effects of Pulsed Pressure Curing on Beef Quality

**DOI:** 10.3390/foods12030656

**Published:** 2023-02-03

**Authors:** Chuang Li, Jiyong Shi, Xiaodong Zhai, Zhikun Yang, Xiaowei Huang, Zhihua Li, Yanxiao Li, Xiaobo Zou

**Affiliations:** 1 School of Food and Biological Engineering, Jiangsu University, Zhenjiang 212013, China; 2 International Joint Research Laboratory of Intelligent Agriculture and Agri-Products Processing, Jiangsu Education Department, Jiangsu University, Zhenjiang 212013, China

**Keywords:** pulsed pressure curing, curing methods, volatile compound, beef, physicochemical properties

## Abstract

The study was proposed to investigate the effects of pulsed pressure curing on the beef absorption of the curing solution, cooking loss, moisture content, centrifugal loss, salt content, sensory attributes, texture, microstructures and volatile compounds. Curing methods include the following four treatments: (1) control group 1—static curing (SC); (2) control group 2—vacuum curing (VC); (3) control group 3—pressurized curing (PC); and (4) treatment group—pulsed pressure curing (PPC). The acquired results revealed that pulsed pressure curing significantly boosts the curing efficiency and moisture content, decreases cooking loss in beef, brightens meat color, and enhances texture compared to static curing, vacuum curing, and pressurized curing. Additionally, centrifugal losses were not impaired, and sensory findings revealed that PPC significantly improved the saltiness of beef. TPA results showed that the springiness and cohesiveness of PPC were greatly increased, and hardness and chewiness were significantly reduced. Moreover, PPC significantly reduced the content of 1-octen-3-ol and 1-hexanol. Scanning electron microscopy (SEM) images documented that pulsed pressure curing can effectively increase the tenderness of beef. This study demonstrates that processed meat product efficiency and sensory attributes should be taken into account when selecting a curing technique, and the PPC technique has an advantage in both areas.

## 1. Introduction

Curing involves soaking food products in a curing solution in order to enhance their sensory and organoleptic qualities. During the curing process, water transfers from foods to curing solution, and salt (NaCl) is transferred due to the concentration difference [1]. There are more ions in the curing solution than in the muscle fiber cells, allowing salt ions to diffuse across cell membranes until equilibrium is attained. The myogenic fibers absorb and hold vast amounts of water as a result of ion concentration changes, while osmosis and capillary mechanisms cause swelling to more than double their initial volume [2]. The curing solution samples often take hours to achieve a certain concentration owing to the restricted permeability of salt in cell membranes. As a result, an effective brining technique that minimizes the brining time and increases the product consistency is the need of the hour [3].

The frequently used techniques in industrial meat processing include pulsed pressure salting, static brining, pulsed discharges technology, high hydrostatic pressure, high hydrodynamic pressure, shock wave treatment, and vacuum tumbling curing [4,5,6,7,8]. The high hydrostatic pressure accelerates diffusion and osmotic processes, while high hydrodynamic pressure causes intense filtration processes along with diffusion and osmotic processes. Previous studies reported that high hydrostatic pressure is better for tender deboned meat, and high hydrodynamic pressure is superior for meat-on-bone or tough meat [5,8]. Pulsed pressure curing is a method of curing in which meat is immersed in a constant curing solution and alternately subjected to air pressure, vacuum, and positive pressure. This method could preserve the raw meat appearance and tissue composition while averting softening and distortion. Moreover, vacuum tumbling can enhance the mechanical activity by raising the meat to the highest point of rotation in a cylindrical drum and dropping it to the lower surface by gravity. The other two curing techniques can hasten curing fluid entrance into meat and shorten curing time and increase meat softness and quality compared to static salting [9,10].

Previously, various studies reported the impact of vacuum curing techniques on pork and chicken [11,12]; however, there are fewer studies on the quality of pulsed pressure-cured beef [13]. Therefore, this study was proposed to ascertain the effect of PPC on the quality of beef. The effects of PPC were evaluated by documenting the changes in the curing absorption rate, cooking loss, texture, and color of beef. The superheated-steam-roasted beef was also subjected to sensory evaluation. Scanning electron microscopy was used to examine the microstructure of the beef. This study can deliver theoretical foundations for the advancement of curing technologies in meat processing facilities.

## 2. Materials and Methods

### 2.1. Materials

The beef sample used in this study was purchased from a local commercial company, and fat was trimmed before the beef was sliced into pieces. The sample was transported to the laboratory using ice cubes to maintain the lower temperature. Analytical grade NaCl was purchased from China Pharmaceutical Group Co., Ltd. (Beijing, China) and used to prepare curing solution.

### 2.2. Sample Preparation and Salting

**Figure 1:**
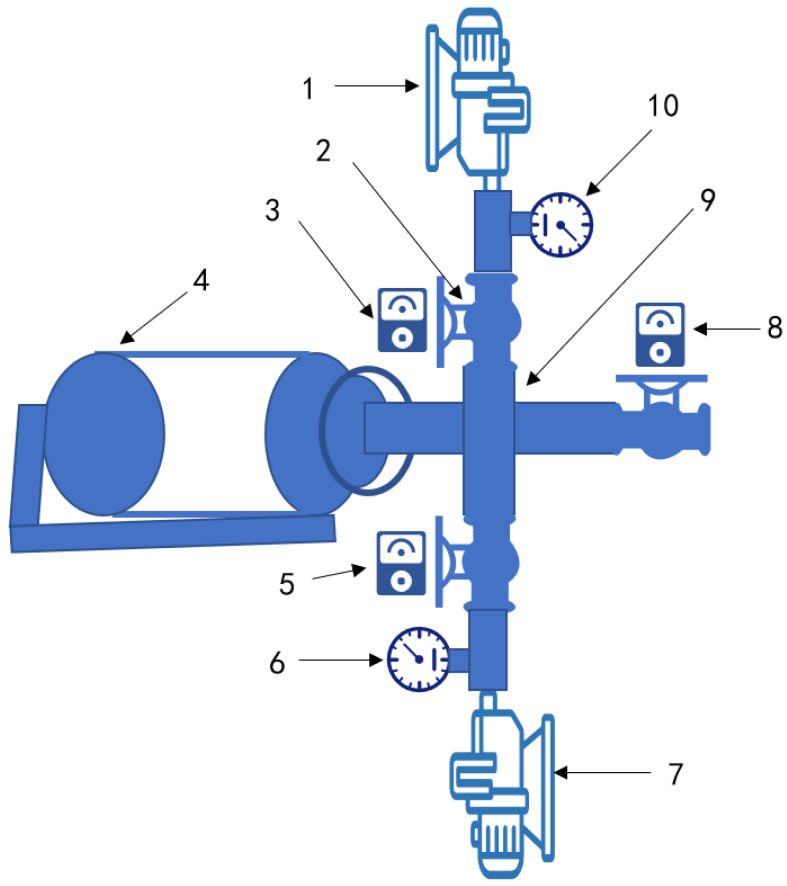
Schematic diagram of the pulsed pressure curing equipment. (1. Booster pump; 2. Electric two-way valve; 3. Positive pressure control module; 4. Pickling container; 5. Negative pressure control module; 6. Negative pressure control table; 7. Vacuum pump; 8. Atmospheric control module; 9. Four-way valve; 10. Positive pressure control table.).

The beef samples were covered in plastic waterproof film and kept at −20 °C for further studies. The samples were initially thawed at 4 °C for 24 h before the experiment. The beef samples were cut into a pieces 4 cm long, 4 cm broad, and 4 cm thick after removing any visible fat and connective tissue. The curing solution (6% sodium chloride) was added to the meat at a rate of 40% of the weight of the meat. The four different therapy approaches were employed for 2 h to cure all the treatment sample as follows: (1) control group 1—take a warm (25 °C) dip in the curing solution (static curing; SC); (2) control group 2—with the vacuum degree of −60 KPa (vacuum curing; VC); (3) control group 3—with the pressure value of 160 KPa (pressurized curing; PC); (4) treatment group—program repeated (10 min at −60 KPa, 10 min at 101 KPa, and 10 min at 160 KPa at room temperature; pulsed pressure curing, PPC). The PPC equipment is a fully automatic variable pressure curing as depicted in Figure 1. The cured beef was washed with running water and then treated using superheated steam at 200 °C for 40 min. Each experiment was independently repeated three times.

### 2.3. Curing Absorption

The curing absorption (CA) was determined using following expression.

CA = 100 × (*W*_0*h*_ − *W_b_*)/*W_b_* (1)

where *W_b_* is the weighed before curing, *W*_0*h*_ is the weight of beef after removing from the curing solution (2 min) [14].

### 2.4. Study of Physical and Chemical Parameters

The pH of the beef samples was determined using a glass pH electrode (Shanghai Lichen Instrument Co., Ltd., Shanghai, China). The salt content was calculated using the Volhard standard titrametric technique [15]. Moisture content of the beef samples was determined using the AOAC Official Method 950.46 (AOAC, 1990). The samples were dried in oven at 103 ± 2 °C for 24 h and calculated using the following equation:

moisture content (%) = 100 × (*W*_1_ − *W*_2_)/*W*_1_ (2)

where *W*_1_ represents the weight of the meat before drying and *W*_2_ represents the weight of the meat after drying.

### 2.5. Cooking Loss

After curing, the beef samples were washed with running water, treated using superheated steam at 200 °C for 40 min, and placed at 4 °C for 12 h. Additionally, filter sheets were used to absorb the water that was present on the sample surface [11]. The cooking loss was calculated using the following expression:

Cooking loss (%) = (*W_b_* − *W_a_*)/*W_b_* × 100 (3)

where *W_b_* and *W_a_* represent the weights of raw and cooked beef samples, respectively.

### 2.6. Determination of Centrifugal Loss

Previously reported method was used to determine the centrifugation loss rate with slight modifications [16]. Briefly, 3 g (*m*_1_) of beef was wrapped in a filter paper and centrifuged at 3000× *g* for 10 min (TG16-WS, Xiangli Scientific Instrument Co., Changsha, China). The sample mass (*m*_2_) was calculated after draining the surface water. The following expression was used to determine the centrifugal loss:

Centrifugal loss (%) = (*m*_1_ − *m*_2_)/*m*_1_ × 100% (4)

where *m*_1_ is the sample mass before centrifugation and *m*_2_ is the sample mass after centrifugation.

### 2.7. Color

The color values (lightness (L*), redness (a*), and yellowness (b*)) were measured using a portable Minolta reflectance colorimeter (Minolta Corp., Ramsey, NJ, USA) and C lighting source [14]. The results are presented as an average of three measurements.

### 2.8. Determination of Texture

The samples were firstly divided into 2 cm × 2 cm × 2 cm pieces and tested for quality indicators such as hardness, chewiness, and elasticity using a texture analyzer (TA. TOUCH, Shanghai Bosin Tech Co., Ltd., Shanghai, China). The reported method is consistent with the study reported previously [17]. The following criteria were employed: 20 g trigger force; P/50 probe type (diameter: 50 mm); 2.0 mm/s pre-measurement speed; 1.0 mm/s measurement speed; 2.0 mm/s post-measurement speed; 50% compression; and 5.0 s between probe measurements.

### 2.9. Sensory Analysis

#### 2.9.1. Trained Analytical Panel

Ten members were selected from a pool of trained panelists who were skilled at sensory evaluation and had a minimum of 2 years of experience in odor and flavor analysis of meat and meat products. Panelists had been recruited, selected, and initially trained under international guidelines (ISO 8586). During a familiarization session, the panelists reviewed an outline of the traits to be assessed, the definitions for each trait, and the techniques to be used for assessment. Panelists were seated in individual ventilated booths under red lights to mask visual differences and were provided with filtered water and crackers as palate cleansers between samples [18]. Three training sessions (30 min for each) and a testing experiment were conducted to let the assessors be familiar with the beef samples. At each session, eight samples comprising two from each experimental group were served monadically. These were randomly distributed to panelists within each session to minimize order and carry-over effects. Panelists evaluated for beef aroma, texture, juiciness, color, saltiness, tenderness, and other flavors. They scored the intensity of each trait on anchored line scales, with marks later converted to a value between 0 and 9.

#### 2.9.2. Consumer Acceptability

The staff members at Jiangsu University were encouraged to participate in this activity, and all the participants were informed of the intention to taste cooked beef for palatability. A total of 40 adult consumers participated and each tasted meat from all four of the experimental groups. Samples were placed onto polystyrene food trays that had been pre-labeled with three-digit random codes. Sample orders were randomized according to a Latin square design to balance for order and carry-over effects [19]. Each sample was assessed for aroma, saltness, tenderness, juiciness, and overall acceptability using a 9-point hedonic scale (1 = dislike extremely to 9 = like extremely) [20]. All procedures and ethics were in agreement with the Declaration of Helsinki. The Ethical Committee of Jiangsu University reviewed the procedures and ethics in advance and stated that no ethics approval was required as an expert panel carried out the evaluation. The study received written informed consent from all participants.

### 2.10. Scanning Electron Microscopy (SEM)

Firstly, the cured meat was cut into cubes (2 mm × 2 mm × 2 mm) perpendicular to the muscle fiber and dried in a freeze-drier after being frozen in liquid nitrogen (Beijing Boyikang Experimental Instrument Co., Ltd., Beijing, China). The freeze-drying time and pressure was 24 h and 4 Pa, respectively. Thereafter, the cubes were coated with gold (1.5 kV, 30 mA, 2 min) in EMITECH K550 equipment, and samples were observed using scanning electron microscopy (JSM-5410, Jeol, Tokyo, Japan) at an acceleration voltage of 20 kV [11].

### 2.11. Volatile Compound Analysis

A previously reported method with slight modification was used for the analysis of volatile compounds [21]. Solid-phase microextraction was used to remove headspace volatile compounds from a 75 μm DVB/CAR/PDMS fiber (SPME). Briefly, 5 g samples were cooked with superheated steam, then the samples were heated at 90 °C for 10 min. The prepared samples were then added to 2 µL of 0.16 mg/mL 2-methyl-3-heptanone in methanol as an internal reference. Thereafter, extraction was carried out at 55 °C for 40 min. The extracted gas was then injected to GC-MS for the analysis of volatile compounds (GCMS-QP2010). The compounds were speculatively identified by comparing fragmentation patterns in EI mass spectra with those recorded by the National Institute of Standards and Technology. Their linear retention indices were then compared to confirm their identity (LRI).

### 2.12. Statistical Analysis

The acquired results were reported as a mean and standard deviation (SD). All the results are the average of three measurements. The physicochemical results were statistically analyzed using SPSS (Statistical Product Service Solutions) 19.0 (SPSS Inc., Chicago, IL, USA). software using a one-way analysis of variance [22]. The Duncan’s multiple range test was used to compare treatment means, and statistically significant differences were indicated by a *p*-value of less than 0.05. Sensory data were analyzed using the principal component analysis (PCA). The PCA analysis was performed using XLSTAT 2019 (Addinsoft, New York, NY, USA), with tasting session as fixed effect and panelist as random effect in the model.

## 3. Results and Discussion

### 3.1. Curing Absorption

**Figure 2:**
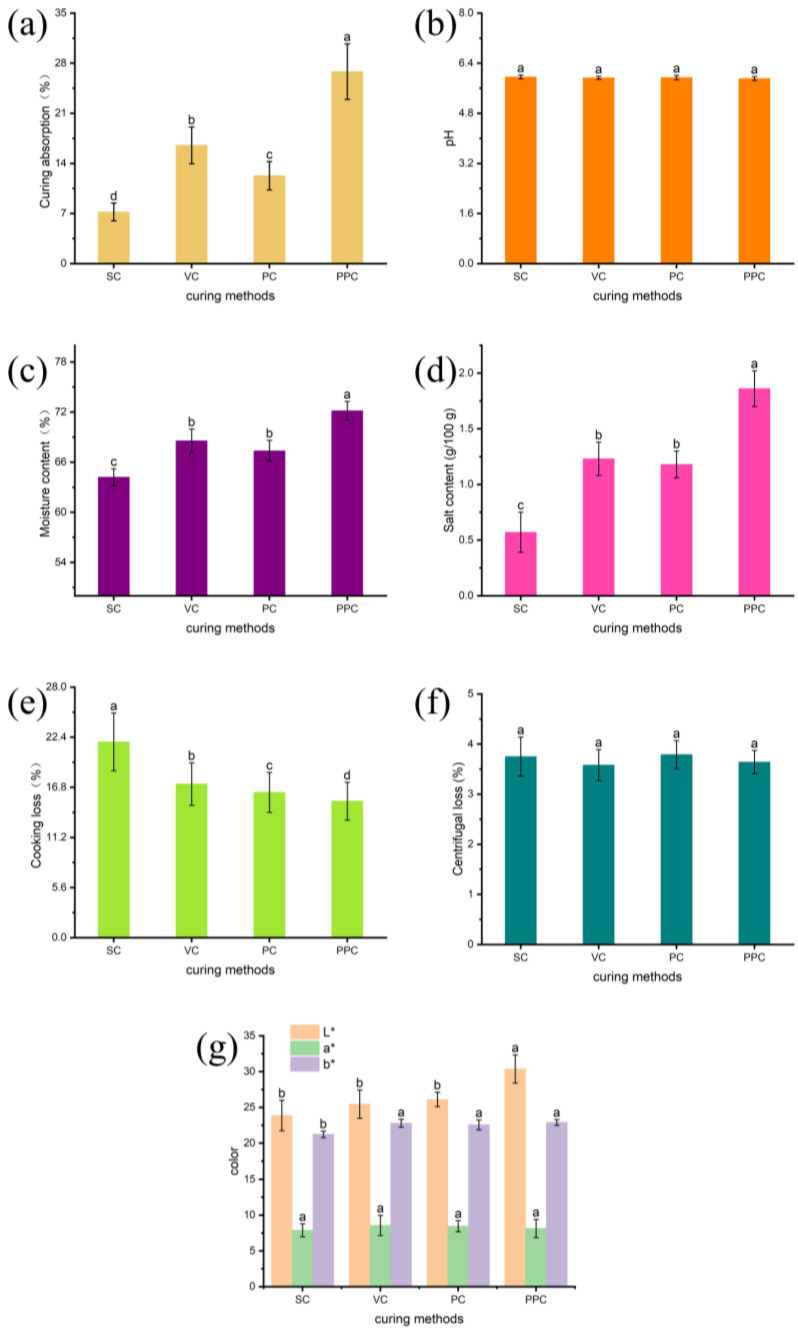
Effects of various curing techniques on the absorption of curing solution (**a**), pH (**b**), moisture content (**c**), salt content (**d**), cooking loss (**e**), centrifugal loss (**f**), and color (**g**) of beef. Different letters (a–d) in the same histogram indicate significant differences (*p* < 0.05).

The curing absorption rate is an important indicator that directly reflects the cured effect of beef. As shown in Figure 2a, the curing absorption rate of the treatment group and three control groups was 26.82%, 7.21%, 16.52%, and 12.26%, respectively. The distinction was appreciably different from the control group (*p* < 0.05). The curing efficiency of the treatment group was noticeably higher than that of the control group. The pulsed pressure curing can effectively improve the curing efficiency because pulsed pressure curing is carried out alternately under the three states of vacuum, normal pressure, and the pressure amplitude changes continuously. According to the mass transfer dynamics, the principle of fluid mechanics and the phenomenon of deformation relaxation, the alternating change of this pressure causes the meat tissue to change and the intercellular space to expand, which effectively promotes the absorption of the salted liquid [23]. When the pulsed pressure curing is in a vacuum state, the structure of the meat expands, and the gas and free-flowing water inside the meat are continuously discharged. When the normal pressure is restored, the salted liquid enters the inside of the meat gap, and when the curing is under pressure, the salted liquid further enters the interior of the meat tissue. The acquired results are in accordance with the findings of Villacís’s research and speed up the migration of solutes while distributing salt uniformly throughout the meat [24]. Conclusively, pulsed pressure curing helps the meat absorb more curing solution and increases the rate at which the curing solution penetrates the meat.

### 3.2. Study of Physical and Chemical Parameters

The pH of beef with different curing treatments is shown in Figure 2b. The pH values of SC, VC, PC, and PPC were 5.96, 5.93, 5.94, and 5.91, respectively. Although the difference between the pH values of the PPC curing therapy and the SC curing treatment in the control group was marginal, no statistical significance was observed (*p* > 0.05). Proteolysis and phosphate diffusion from the curing solution into the meat were the major causes of the pH value dropping throughout the salting process [10].

The moisture content of beef with different curing treatments is presented in Figure 2c. The moisture content values of SC, VC, PC, and PPC were 64.19%, 68.56%, 67.36%, and 72.16%, respectively. When compared to the control groups of SC curing treatment, the moisture content in the PPC curing treatment was considerably greater (*p* < 0.05). This could be attributed to the pressure pulses driving force, which may have encouraged moisture penetration into the meat. This finding was in line with previous study that showed salting significantly increased the ability of pork mince and turkey breast to retain water [25]. Increased water retention capacity is associated with transverse muscle fiber stretch. This is because salt increases the electrostatic repulsion between muscle fiber filaments, causing the filament lattice to expand and trap water [26], implying that PPC curing procedure might substantially enhance meat pickling speed.

The salt content of beef with different curing treatments is shown in Figure 2d. The salt content values of SC, VC, PC, and PPC were 0.57 g/100 g, 1.23 g/100 g, 1.18 g/100 g, and 1.86 g/100 g, respectively. The beef under PPC treatment had the greatest salt concentration, indicating that PPC was successful in promoting NaCl penetration. These findings are in line with the outcomes found by Jin et al. [10], who investigated pork cured with help from pulse pressure. 

According to the findings, the curing pork ion NaCl level was almost twice as high under pulse pressure as it was at normal pressure. According to Wang et al. [27], the salt concentration of the lamb during pulse vacuum salting from 1.5 to 6 h was 1% greater than during atmospheric salting. The expanded muscle structure under vacuum during PPC treatment may have raised the salt content because it dissolves the free gas in the interior tissues and speeds up the diffusion of the therapeutic liquid into the muscle. When the vacuum is released and the atmosphere is once again in contact with the muscle, the healing liquid quickly penetrates via the tissue pores, speeding the migration of salt and raising the salt concentration [27].

### 3.3. Cooking Loss

Cooking loss is one of the most important indicators to evaluate the water retention capacity of meat. Evidently from Figure 2e, the impact of various curing techniques on the cooking loss of beef can be observed. The cooking losses of SC, VC, PC, and PPC were 21.87%, 17.16%, 16.23%, and 15.25%, respectively. The results showed that the PPC treatment in the experimental group could significantly reduce the cooking loss of beef (*p* < 0.05) compared to the control group (SC, VC, and PC). Pulsed pressure curing promotes the dissolution of rich salt-soluble proteins in the meat surface layer, preventing the outflow of water, improving water retention and reducing cooking losses [28]. Overall, the cooking loss of beef can be considerably decreased by PPC treatment.

### 3.4. Centrifugal Loss

The water-holding capacity (WHC) of meat is typically evaluated by determining cooking loss and centrifugal loss, where centrifugal loss is the water lost from cooked meat samples. Evidently from Figure 2f, the centrifugal loss values of SC, VC, PC, and PPC were 3.75%, 3.58%, 3.79%, and 3.64%, respectively. No noticeable difference was observed in centrifugal loss between the beef with various curing methods.

### 3.5. Color

The impact of curing techniques on the color value of meat is presented in Figure 2g. PPC had the ability to dramatically raise the L* value of beef in comparison to SC, VC, and PC (*p* < 0.05). The alternating pressure changes the meat tissue, destroys myofibroblasts, and dissolves myoglobin. It increases the meat brightness value while decreasing the myoglobin in beef [29]. The various curing methods did not substantially differ in the color characteristics a* and b* (*p* > 0.05). The effect of pulsed pressure curing on meat color brightness is hence very negligible.

### 3.6. Texture

The textural profile analysis (TPA) results of beef with different curing treatments are presented in Table 1. The springiness and cohesiveness of the PPC curing treatment greatly enhanced (*p* < 0.05) compared to the SC curing treatment, whereas the hardness and chewiness were dramatically decreased. The current study suggests that the PPC curing treatment ability to tenderize beef may be connected to the influence that pressure has on the muscles level of contraction. The tenderizing effect might be attributed to the myofibrillar structure being broken as a result of the combined effects of the treatments pressure and muscular contraction, which would force myosin filaments of highly tensed muscle into Z discs [30]. Additionally, PPC treatment reduced the meat hardness compared to the SC curing treatment. This effect might potentially be attributable to the activation of the cathepsins and calpains [31]. The effort needed to chew a sample to a consistent condition of swallowing is referred to as chewiness, whereas a samples ability to regain its previous shape after deformation is referred to as springiness [32]. Juiciness and softness are indicated by springiness and chewiness respectively. The relationship between chewiness and hardness was strong. All of these TPA results suggested that PPC treatment might successfully enhance the textural quality of beef samples.

### 3.7. Sensory Evaluation: Trained and Consumer Panel

**Figure 3:**
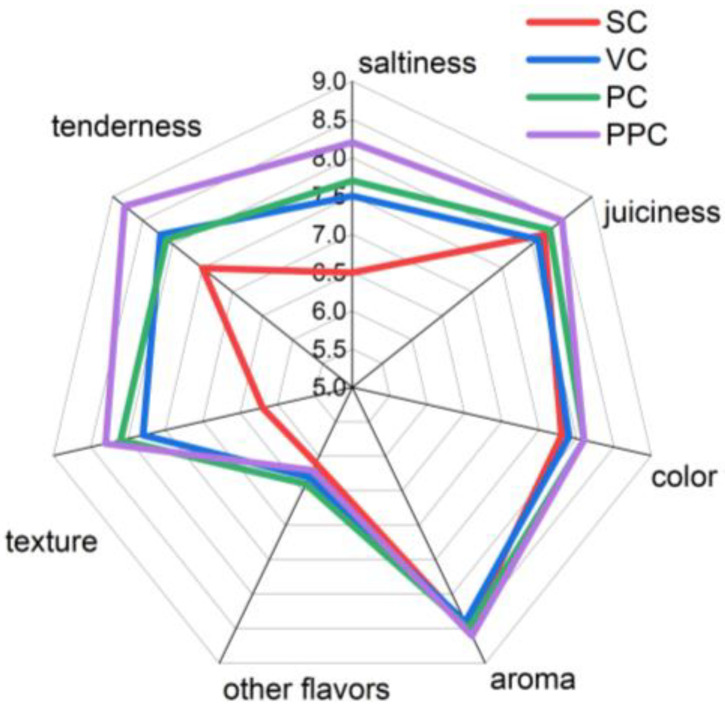
Effect of beef with different curing treatments on the sensory attributes as evaluated by a trained panel. (SC = static curing, VC = vacuum curing, PC = pressurized curing, PPC = pulsed pressure curing).

Sensory attributes of beef with different curing treatments were assessed by a trained panel, and their results are presented in Figure 3. The findings revealed that there was no apparent difference in the aroma, juiciness, or color of beef treated using various curing techniques (*p* > 0.05). PPC could significantly improve the texture of beef owing to the changes in meat tissue caused by the alternating of pressure, which broke down the muscle fibers and decreased the sheer force of beef. There were significant differences in overall texture among the treatments with different curing methods (*p* < 0.05), and this modification could tenderize the meat [33]. There were significant differences (static curing, vacuum curing, pressurized curing, pulsed pressure curing) in the saltiness of beef treated with different curing methods (*p* < 0.05), and PPC could significantly improve the saltiness of beef, which was due to the changes in the meat tissue and the expansion of intercellular spaces due to the alternating changes of pressure, which effectively promoted the curing solution absorption [24]. Results from consumer acceptability tests of the same meats also indicated no difference in the aroma and juiciness of beef treated using various curing techniques (Table 2). PPC had the best overall approval rating. In general, PPC treatment improved the flavor of the meat and was worthy of future investigation.

### 3.8. Scanning Electron Microscopy

**Figure 4:**
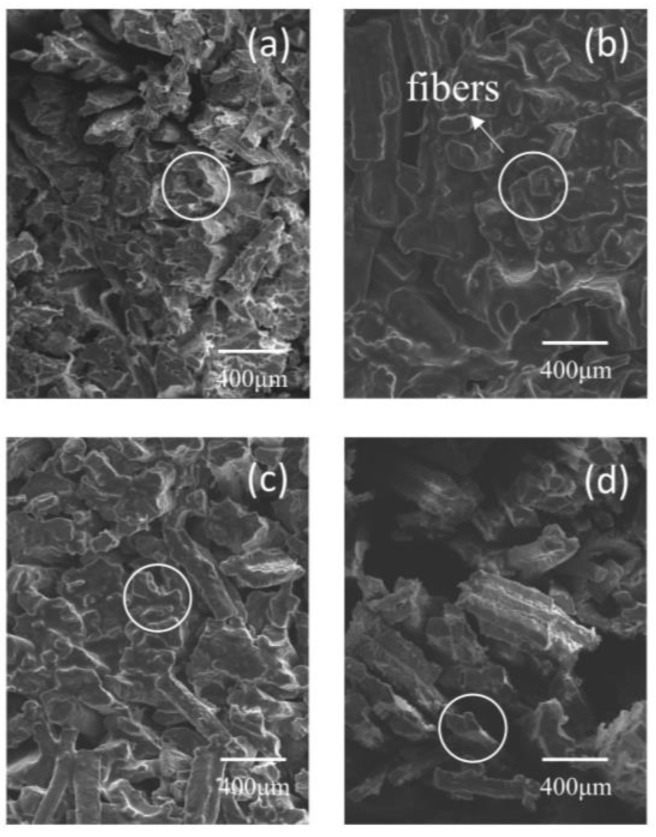
Effects of different curing methods on muscle fiber of beef. (**a**) SC; (**b**) VC; (**c**) PC; (**d**) PPC. SC = static curing, VC = vacuum curing, PC = pressurized curing, PPC = pulsed pressure curing.

The effect of SC, VC, PC, and PPC curing procedures on the microstructure of beef are depicted in Figure 4a–d. Figure 4a shows a rather compact fiber structure after SC treatment. Evidently from Figure 4d, the PPC treatment revealed a distinct space between the neighboring muscle bundles of beef tissue. The pulsed pressure curing is carried out alternately under the three states of vacuum, normal pressure, and pressure, and the pressure amplitude changes continuously. According to the mass transfer dynamics, the principle of fluid mechanics and the phenomenon of deformation relaxation, the alternating change of this pressure causes the meat tissue to change and the intercellular space to expand, and this reduced cooking waste and enhanced the brining effect, including curing solution absorption and tenderness [24].

### 3.9. Volatile Compound

These volatile substances comprised acids, alcohols, aldehydes, hydrocarbons, esters, furan, and ketones and, determined by their chemical structures, are documented in Table 3. The three most prevalent volatile flavoring compounds in beef were alcohols, aldehydes, and hydrocarbons [33]. The acid, ester, hydrocarbon, and furan contents of the beef through various curing procedures were unaffected. The levels of ketones, aldehydes, and alcohol were considerably greater in the PPC curing treatment groups compared to the SC curing treatment (*p* < 0.05).

The primary byproducts of fatty acid breakdown are aldehydes. They greatly affect the aroma of meat items, have a low odor threshold, and have a fatty smell [34]. Aldehydes were the most prevalent volatile flavoring substances in meat, as reported previously [21,35]. Benzaldehyde was the sole identified aromatic aldehyde, whereas the majority of aldehydes were aliphatic [21]. Hexanal was the predominant aldehyde in beef flesh as reported in the previous literature. This was in line with Jin’s findings that the PPC curing process encouraged the oxidation of fat to produce volatile chemicals [36].

Ketones are produced by either the Strecker degradation of amino acids or the oxidation of unsaturated fatty acids. Volatile ketones have a fruity or creamy taste. Some ketones serve as significant catalysts in the production of heterocyclic aroma molecules [37]. In the current investigation, substantially fewer kinds and lower amounts of ketones than aldehydes were found in beef flesh. The similar findings were also previously documented for pork samples.

Alcohols are produced by the oxidation of fatty acids and help to establish the taste of cooked meat [38]. The decreased synthesis of 1-octen-3-ol and 1-hexanol during curing may be advantageous for the odor of processed beef products [39] because both substances are thought to be significant contributors to off-flavors. Conclusively, PPC curing process can decrease the production of off tastes when compared to other curing techniques.

### 3.10. Multivariate Analysis of Sensory Attributes

**Figure 5:**
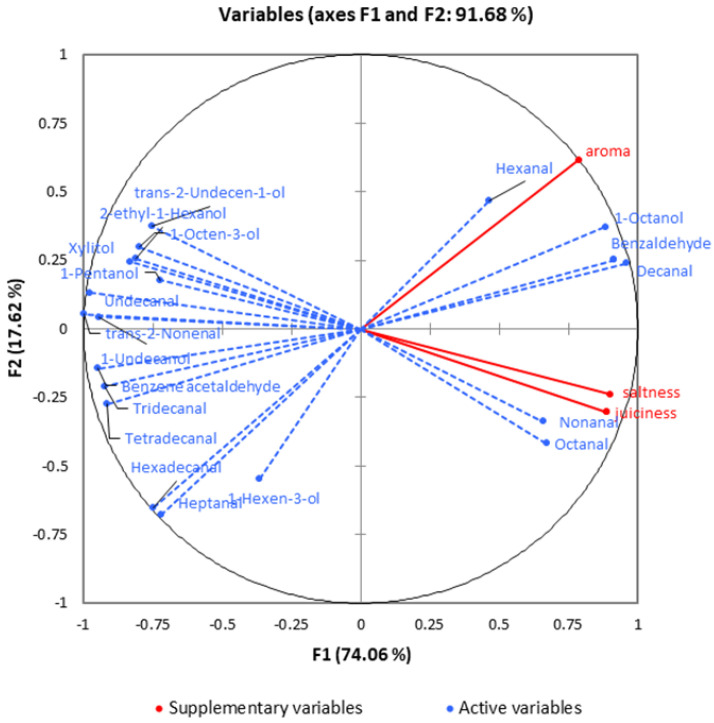
PCA results of the main volatile compounds and sensory attributes. Red solid lines represent sensory attributes, blue dashed lines represent volatile compounds.

Sensory attributes influenced the overall liking of consumers of different beef samples. The PCA was applied to explore the influence of main volatile compounds on the sensory attributes scores. In this study, 20 volatile compounds which had significant differences among different beef samples were selected as the main volatile compounds for the PCA analysis. The volatile compounds and the sensory attributes were regarded as active and supplementary variables, respectively. Evidently from Figure 5, 1−Octanol and aldehydes (hexanal, octanal, and nonanal) were positively correlated with the aroma and juiciness. These findings were consistent with the conclusion that aldehydes compounds played an important role in improving the meaty odor of meat products [40].

## 4. Conclusions

The current study was employed to examine the quality of beef under various curing techniques. The findings revealed that PPC curing method successfully increased the meat color brightness, decreased the cooking loss, and improved the curing efficiency compared to the control group. The curing absorption rate reached a maximum of 26.82% and cooking loss was as low as 15.25%. PPC treatment also significantly improved the moisture content (72.16%) and salt content (1.86 g/100 g) of beef. In addition, centrifugal losses were not impaired in the PPC treatment compared to the control group. The sensory findings showed that PPC treatment significantly enhanced the saltiness of beef and maintained the desired sensory properties. TPA results indicated that the springiness and cohesiveness of PPC were greatly improved, and the hardness and chewiness were significantly reduced. Moreover, PPC significantly reduced the content of 1-octen-3-ol and 1-hexanol content. Scanning electron microscopy images showed that PPC curing can effectively increase the tenderness of meat. PPC curing treatment can greatly reduce the curing time and improve the product quality, benefiting both the processors and consumers by lowering production cost and increasing productivity. The shortening of the curing time and improvement of product quality are of great value for meat processors.

## Figures and Tables

**Table 1:** Texture of beef with different curing treatments.

Curing Methods	Hardness (g)	Springiness (mm)	Chewiness (g)	Cohesiveness
SC	5112.74 ± 236.78 ^a^	0.76 ± 0.04 ^b^	1448.58 ± 121.21 ^a^	0.54 ± 0.02 ^c^
VC	4125.45 ± 212.67 ^c^	0.76 ± 0.03 ^b^	1227.26 ± 113.37 ^b^	0.59 ± 0.02 ^b^
PC	4556.94 ± 196 ^b^	0.78 ± 0.04 ^b^	1328.43 ± 98.51 ^b^	0.63 ± 0.02 ^b^
PPC	3892.97 ± 158.77 ^c^	0.85 ± 0.05 ^a^	987.56 ± 57.51 ^c^	0.67 ± 0.04 ^a^

Values in the same column followed by different letters differed significantly (*p* < 0.05). SC = static curing, VC = vacuum curing, PC = pressurized curing, PPC = pulsed pressure curing.

**Table 2:** Consumer acceptability tests of beef with different curing treatments by a cohort of adult consumers using a 9-point hedonic scale from 1 = dislike extremely to 9 = like extremely.

Acceptability	SC	VC	PC	PPC
aroma	8.4 ± 0.3 ^a^	8.3 ± 0.3 ^a^	8.4 ± 0.2 ^a^	8.5 ± 0.3 ^a^
saltness	6.4 ± 0.2 ^c^	7.3 ± 0.3 ^b^	7.4 ± 0.2 ^b^	8.4 ± 0.2 ^a^
tenderness	7.4 ± 0.3 ^c^	8.2 ± 0.2 ^b^	8.1 ± 0.2 ^b^	8.7 ± 0.2 ^a^
juiciness	7.8 ± 0.3 ^b^	8.0 ± 0.2 ^b^	7.9 ± 0.2 ^b^	8.0 ± 0.2 ^a^
overall	7.3 ± 0.1 ^c^	7.9 ± 0.2 ^b^	7.8 ± 0.2 ^b^	8.4 ± 0.2 ^a^

Values in the same row followed by different letters differed significantly (*p* < 0.05). SC = static curing, VC = vacuum curing, PC = pressurized curing, PPC = pulsed pressure curing.

**Table 3:** Contents of volatile compounds on beef with different curing treatments.

No	Volatile Compounds	Content (μg·kg^−1^)			
		SC	VC	PC	PPC
1	1-Hexanol	0.82 ± 0.08 ^a^	0.54 ± 0.02 ^b^	0.55 ± 0.02 ^b^	0.42 ± 0.03 ^c^
2	1-Octen-3-ol	3.76 ± 0.83 ^a^	2.63 ± 0.82 ^b^	2.15 ± 0.05 ^c^	2.13 ± 0.07 ^c^
3	2-ethyl-1-Hexanol	9.87 ± 0.82 ^b^	12.06 ± 1.01 ^a^	12.16 ± 1.22 ^a^	12.24 ± 1.61 ^a^
4	Xylitol	6.65 ± 1.03 ^b^	6.41 ± 0.92 ^b^	8.64 ± 0.82 ^a^	8.44 ± 0.62 ^a^
5	1-Pentanol	5.95 ± 1.02 ^c^	9.71 ± 1.12 ^b^	9.95 ± 1.05 ^b^	12.95 ± 1.92 ^a^
6	1-Hexen-3-ol	9.16 ± 1.23 ^b^	13.16 ± 1.82 ^a^	8.86 ± 1.65 ^b^	13.77 ± 1.54 ^a^
7	1-Octanol	8.11 ± 0.82 ^b^	8.52 ± 0.69 ^b^	8.23 ± 0.57 ^b^	12.26 ± 0.62 ^a^
8	1-Undecanol	5.77 ± 0.83 ^b^	5.37 ± 0.96 ^b^	8.94 ± 1.32 ^a^	9.57 ± 1.53 ^a^
9	trans-2-Undecen-1-ol	5.59 ± 0.58 ^b^	8.56 ± 0.68 ^a^	8.84 ± 0.65 ^a^	8.67 ± 0.98 ^a^
10	3,7,11-trimethyl-1-Dodecanol	6.11 ± 0.67 ^a^	6.52 ± 0.86 ^a^	6.28 ± 0.69 ^a^	6.09 ± 0.89 ^a^
11	Hexanal	117.46 ± 9.51 ^c^	137.36 ± 8.62 ^b^	139.61 ± 9.12 ^b^	167.24 ± 10.23 ^a^
12	Heptanal	28.69 ± 2.2 ^b^	29.71 ± 2.1 ^b^	28.66 ± 1.45 ^b^	35.74 ± 3.15 ^a^
13	Benzaldehyde	141.66 ± 6.58 ^b^	167.82 ± 9.52 ^a^	169.81 ± 8.46 ^a^	172.58 ± 8.45 ^a^
14	Octanal	58.82 ± 4.1 ^b^	60.07 ± 3.12 ^b^	67.92 ± 4.15 ^a^	68.93 ± 5.12 ^a^
15	Benzene acetaldehyde	36.98 ± 1.8 ^c^	43.36 ± 2.02 ^b^	45.1 ± 3.45 ^b^	51.43 ± 3.48 ^a^
16	Nonanal	170.33 ± 7.52 ^b^	196.62 ± 9.54 ^a^	199.76 ± 8.41 ^a^	202.92 ± 10.56 ^a^
17	trans-2-Nonenal	47.4 ± 3.1 ^b^	49.59 ± 4.31 ^b^	55.44 ± 3.81 ^a^	57.59 ± 4.21 ^a^
18	Decanal	60.94 ± 4.5 ^b^	69.67 ± 3.2 ^a^	68.37 ± 4.1 ^a^	70.06 ± 3.02 ^a^
19	Undecanal	56.32 ± 5.82 ^b^	54.48 ± 6.32 ^b^	54.59 ± 7.41 ^b^	71.86 ± 6.22 ^a^
20	Dodecanal	23.74 ± 4.9 ^a^	22.8 ± 5.8 ^a^	23.76 ± 6.03 ^a^	21.59 ± 6.61 ^a^
21	Tridecanal	34.46 ± 5.3 ^b^	34.42 ± 5.48 ^b^	33.33 ± 6.14 ^b^	44.47 ± 3.45 ^a^
22	Tetradecanal	68.08 ± 8.1 ^b^	93.94 ± 7.47 ^a^	72.5 ± 7.26 ^b^	96.67 ± 8.75 ^a^
23	Pentadecanal	79.44 ± 4.8 ^a^	81.99 ± 5.58 ^a^	82.72 ± 5.48 ^a^	83.1 ± 6.46 ^a^
24	Hexadecanal	104.16 ± 2.5 ^c^	124.12 ± 3.58 ^b^	123.83 ± 3.47 ^b^	135.09 ± 3.74 ^a^
25	Acetophenone	24.34 ± 3.11 ^c^	34.89 ± 4.41 ^b^	32.15 ± 4.78 ^b^	51.8 ± 5.44 ^a^
26	2-Nonanone	42.27 ± 5.46 ^b^	55.36 ± 8.11 ^a^	59.47 ± 7.46 ^a^	56.54 ± 6.12 ^a^
27	Geranyl acetone	43.34 ± 5.15 ^b^	46.88 ± 7.45 ^b^	48.93 ± 6.78 ^b^	69.58 ± 7.98 ^a^
28	2,5-Octanedione	32.12 ± 4.02 ^b^	35.91 ± 5.32 ^b^	31.23 ± 4.26 ^b^	46.74 ± 6.49 ^a^
29	3-methyl-Butanoic acid	102.26 ± 10.13 ^a^	106.63 ± 8.52 ^a^	103.38 ± 9.44 ^a^	110.18 ± 12.14 ^a^
30	2-methyl-Butanoic acid	89.82 ± 8.01 ^a^	90.41 ± 5.11 ^a^	87.21 ± 9.61 ^a^	92.98 ± 8.71 ^a^
31	2,2-dimethylpropyl acetate	46.96 ± 5.16 ^a^	47.05 ± 3.12 ^a^	45.89 ± 4.12 ^a^	45.83 ± 6.26 ^a^
32	n-Propyl propionate	38.63 ± 6.12 ^a^	39.45 ± 5.87 ^a^	36.11 ± 7.14 ^a^	39.65 ± 5.37 ^a^
33	Ethyl caprylate	25.97 ± 3.65 ^a^	24.71 ± 3.97 ^a^	27.28 ± 2.89 ^a^	26.42 ± 2.15 ^a^
34	Methyl nonoate	36.99 ± 3.97 ^a^	39.45 ± 3.24 ^a^	37.14 ± 2.77 ^a^	38.85 ± 3.45 ^a^
35	Methyl tetradecanoate	44.34 ± 5.46 ^a^	43.45 ± 3.11 ^a^	41.87 ± 5.68 ^a^	45.46 ± 3.62 ^a^
36	Dodecane	79.17 ± 7.43 ^a^	78.22 ± 9.87 ^a^	81.47 ± 8.86 ^a^	80.79 ± 8.25 ^a^
37	Tridecane	78.82 ± 7.89 ^a^	81.46 ± 8.86 ^a^	82.78 ± 9.23 ^a^	79.85 ± 6.85 ^a^
38	2-cyclohexyl-Dodecane	52.32 ± 2.41 ^b^	58.48 ± 2.41 ^a^	57.98 ± 3.96 ^a^	59.78 ± 2.98 ^a^
39	Tetradecane	60.41 ± 5.87 ^a^	62.45 ± 4.65 ^a^	60.54 ± 4.58 ^a^	61.42 ± 3.87 ^a^
40	1-Pentadecene	53.54 ± 4.86 ^a^	51.13 ± 3.65 ^a^	54.54 ± 2.85 ^a^	52.89 ± 3.89 ^a^
41	Pentadecane	66 ± 7.79 ^a^	61.14 ± 9.76 ^a^	62.46 ± 6.85 ^a^	64.78 ± 6.46 ^a^
42	Heptadecane	36.64 ± 7.65 ^a^	31.46 ± 6.34 ^a^	32.74 ± 5.82 ^a^	34.12 ± 6.18 ^a^
43	2-Methyl-3-furanthiol	22.98 ± 3.84 ^a^	23.64 ± 3.68 ^a^	21.45 ± 2.75 ^a^	21.28 ± 2.54 ^a^
44	2-pentyl-Furan	18.64 ± 3.58 ^a^	16.14 ± 4.38 ^a^	17.24 ± 3.89 ^a^	16.37 ± 3.61 ^a^

a–c: Means within the same row with different superscript showing significant differences (*p* < 0.05). SC = static curing, VC = vacuum curing, PC = pressurized curing, PPC = pulsed pressure curing.

## Data Availability

The data presented in this study are available on request from the corresponding author.
